# A Nonsense Mitochondrial DNA Mutation Associates with Dysfunction of HIF1*α* in a Von Hippel-Lindau Renal Oncocytoma

**DOI:** 10.1155/2019/8069583

**Published:** 2019-01-09

**Authors:** Monica De Luise, Vito Guarnieri, Claudio Ceccarelli, Leonardo D'Agruma, Anna Maria Porcelli, Giuseppe Gasparre

**Affiliations:** ^1^Department of Medical and Surgical Sciences (DIMEC), Unit of Medical Genetics, University of Bologna Medical School, Bologna 40138, Italy; ^2^Division of Medical Genetics, Fondazione IRCCS Casa Sollievo della Sofferenza, San Giovanni Rotondo 71013, Italy; ^3^Department of Experimental, Diagnostic, and Specialty Medicine (DIMES), University of Bologna Medical School, Bologna 40138, Italy; ^4^Department of Pharmacy and Biotechnology (FABIT), University of Bologna, Bologna 40128, Italy; ^5^Center for Applied Biomedical Research (CRBA), University of Bologna-S. Orsola Hospital, Bologna 40138, Italy

## Abstract

The Von Hippel-Lindau (VHL) syndrome has been rarely associated with renal oncocytomas, and tumors usually show HIF1*α* chronic stabilization. By contrast, oncocytomas mainly associated with respiratory chain (RC) defects due to severe mitochondrial DNA (mtDNA) mutations are incapable of stabilizing HIF1*α*, since oxygen consumption by the RC is dramatically diminished and prolylhydroxylase activity is increased by *α*-ketoglutarate accumulation following Krebs cycle slowdown. Here, we investigate the cooccurrence of a pseudohypoxic condition with oncocytic transformation in a case of VHL-associated renal oncocytoma. While HIF1*α* was abundant in nuclei concordantly with defects in VHL, negative staining of its targets carbonic anhydrase IX (CAIX) and glucose transporter GLUT1, usually overexpressed in VHL-associated neoplasms, suggested HIF1*α* to be present in its inactive (hydroxylated) form. MtDNA sequencing and immunohistochemistry analyses revealed a *MT-CO1* stop-gain mutation and cytochrome c oxidase loss. We suggest that a mitochondrial respiration impairment may lead to hyperhydroxylation of the transcription factor, which we confirmed by specific staining of hydroxylated HIF1*α*. Such inactive form hence accumulated in the VHL-deficient tumor, where it may contribute to the benign nature of the neoplasm. We propose that the protumorigenic role of HIF1*α* in VHL cancers may be blunted through drugs inhibiting mitochondrial respiratory complexes, such as metformin.

## 1. Introduction

Von Hippel-Lindau (VHL) disease is a genetic condition characterized by the predisposition to develop tumors of the central nervous system, such as hemangioblastoma of the cerebellum, in addition to clear cell Renal Cell Cancer (ccRCC). Pheochromocytoma and pancreatic cancer have also been reported in patients harboring germ line *VHL* mutations [1]. A common molecular feature shared by such neoplasms is the chronic stabilization of the Hypoxia-Inducible Factor 1*α* (HIF1*α*) even in normoxia (pseudohypoxia). The tumor suppressor *VHL* encodes a ubiquitin ligase enzyme that presides to constant HIF1*α* degradation in the presence of molecular oxygen, when the transcription factor is hydroxylated by prolylhydroxylase domain-containing (PHD) enzymes for VHL recognition and proteasome degradation [2]. A lack of VHL leads to HIF1*α* activation sustaining a protumorigenic metabolic reprogramming towards Warburg's glycolytic shift, as well as a promotion of neoangiogenesis, since HIF1*α* positively regulates both glycolytic enzymes and the vascular endothelial growth factor (VEGF). Such protumorigenic changes, on the contrary, do not occur in benign sporadic kidney oncocytomas, i.e., renal tumors characterized by cells with a marked mitochondrial hyperplasia and a scarce vascularization. This is due to the inability of the tumor to stabilize HIF1*α* following the occurrence of pathogenic mitochondrial DNA (mtDNA) mutations [3], a distinctive hallmark of renal oncocytoma [4]. Here, we report for the first time a case of VHL-associated renal oncocytoma whose mtDNA genetic hallmark, a highly pathogenic mutation in respiratory Complex IV (CIV), was associated with the unique phenotype of undegraded, hyperhydroxylated, and dysfunctional HIF1*α*, suggesting that mtDNA mutations are potent modifiers in cancer.

## 2. Materials and Methods

### 2.1. Mitochondrial DNA Sequencing, Haematoxylin-Eosin Staining, and Immunohistochemical Analysis

Analyses were performed on FFPE sections of the proband's renal oncocytoma. 10 *μ*m-thick slides were obtained for total DNA extraction with a commercial kit (Promega), and whole mtDNA sequencing and analysis were performed as previously described [5]. 4 μm-thick FFPE serial sections were used for haematoxylin-eosin staining (H&E) and immunohistochemical (IHC) analysis. The following primary antibodies were applied: mouse monoclonal anti-mitochondrial COXI (clone 1D6E1A8, Abcam Ltd., UK) diluted 1 : 800; mouse monoclonal anti-HIF1*α* (clone H1alpha67, Thermo Fisher Scientific, USA) diluted 1 : 600; rabbit monoclonal anti-hydroxy-HIF-1*α* (clone D43B5, Cell Signaling Technology, USA) diluted 1 : 400; and mouse monoclonal anti-OxPhos Complex V, subunit D (clone 7F9, Invitrogen Co., CA, USA) diluted 1 : 1000. Sections were dewaxed, rehydrated, and subjected to antigen retrieval treatment in a water bath at 98.5°C using citrate buffer pH 6.0 (40 min) for anti-HIF1*α* or Tris-EDTA buffer pH 9.0 (20 min) for anti-COXI and anti-Complex V, subunit D. Endogenous peroxidase activity was inhibited using a 0.5% H_2_O_2_ solution in methanol for 20 min at room temperature. Sections were incubated overnight at 4°C and then processed using a nonbiotin-amplified method (Novolink, Novocastra) according to the manufacturer's protocols. The immunologic reaction was developed using a 3,3′-diaminobenzidine (DAB)/H_2_O_2_ PBS pH 7.2-7.4 solution for 10 min. Sections were then washed in distilled water, counterstained in Harris haematoxylin, dehydrated, and mounted with Bio Mount HM (Bio Optica, Milan Italy). The immunological reaction for anti-CAIX (clone TH-22, Novocastra, UK) diluted 1 : 100 and rabbit polyclonal anti-GLUT1 (Cell Marque, USA) RTU was conducted in a BenchMark ULTRA autostainer (Ventana Medical Systems, USA). Sections were retrieved with Ultra CC1 at 95°C (CAIX—32 min, GLUT1—24 min) and incubated at 36°C for 28 min with the proper antibody. The immunologic reaction was visualized using an OptiView DAB detection kit following the manual instructions.

### 2.2. Immunohistochemical Quantification

The quantitative score (QS) for each protein in control and tumor tissue was evaluated at a magnification of 200x as previously reported [6]. The QS is then expressed as the product of the percentage of positive cells (P) and the staining intensity (I). The final QS is a value within the range of 0 (negative staining) and 12 (strong positive staining).

### 2.3. Ethics Approval and Consent to Participate

The patient was enrolled in the TRANSMIT study (protocol number 26/2009/U/Tess 03/03/2009) approved by the local ethical committee at S. Orsola Hospital, Bologna.

## 3. Results

### 3.1. Case Histology Reveals a Typical Renal Oncocytoma

**Figure 1:**
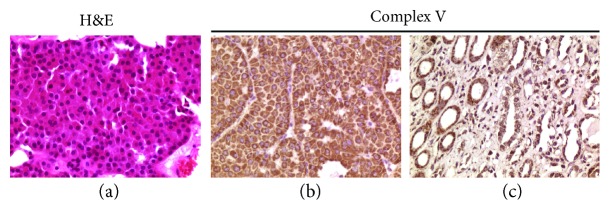
H&E staining and IHC characterization of the renal oncocytoma from the patient carrying the germline *VHL* deletion. (a) H&E staining of tumor tissue. (b, c) IHC for Complex V, subunit D in the renal oncocytoma (b) and in a normal kidney used as control (c), both with a QS of 8 (*P* = 4, *I* = 2). Magnification for all IHC images: 200x.

The case was retrieved from a previous study in which a 40-year-old male had been diagnosed with hemangioblastoma of the cerebellum and renal oncocytoma with a germline *VHL* mutation [7]. The patient's renal tumor was in fact revealed to be a typical oncocytoma located in the lower pole of the right kidney visualized with echography as a well-circumscribed nodule. Macroscopically, the neoplasm measured 3.5 cm across, displayed neat borders, and had a brownish color in the cut surface. The case was brought to our attention with the aim to understand how a pseudohypoxic condition may coexist with the oncocytic phenotype, whose molecular signature is the occurrence of mtDNA mutations leading to HIF1*α* chronic destabilization [3]. Genetic analyses had previously revealed that the patient harbored an 11 bp duplication in the promoter region of *VHL*, whose functional investigation showed an impaired gene expression of the mutated allele, hence explaining the VHL syndromic phenotype of the subject [7]. Histologically, the tumor was constituted by cells of similar size arranged along regular trabeculae. Cells showed round and regular nuclei. Mitoses were rare. No necrotic areas were seen. Almost all tumor cells displayed intense and diffuse eosinophilic cytoplasmic granularity (Figure 1(a)) consequent to mitochondrial overload as observed with the positive anti-OxPhos antibody (Figures 1(b)-1(c)).

### 3.2. A Peculiar HIF1*α* Dysfunction Occurs within the Renal Oncocytoma

**Figure 2:**
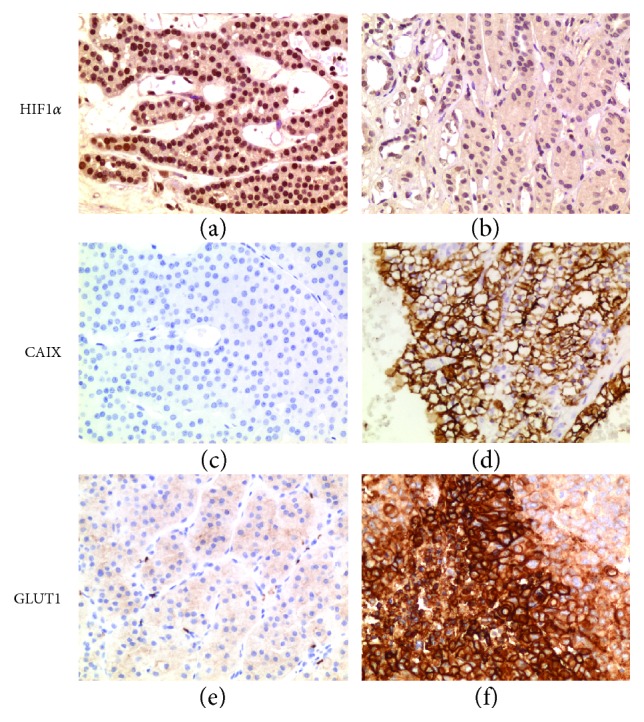
IHC analysis of HIF1*α* and its targets. (a, b) IHC for HIF1*α* in the renal oncocytoma (a) with a QS of 12 (*P* = 4, *I* = 3) and in a normal kidney used as control (b) with a QS of 1 (*P* = 1, *I* = 1). (c, d) IHC for CAIX in the renal oncocytoma (c) with a QS of 0 (*P* = 0, *I* = 0) and in a clear cell renal cell carcinoma used as positive control (d) with a QS of 10 (*P* = 4, *I* = 2.5). (e, f) IHC of GLUT1 in the renal oncocytoma (e) with a QS of 0 (*P* = 0, *I* = 1) and in a hypoxic area of a neuroendocrine carcinoma of the ovary used as positive control (f) with a QS of 12 (*P* = 4, *I* = 3). Magnification for all IHC images: 200x.

Since VHL-associated tumors usually show a functional HIF1 even in normoxia, we proceeded to perform IHC for the HIF1*α* subunit, which we expected to be positive, as the effect of *VHL* loss of function mutations is HIF1*α* chronic stabilization due to lack of proteasomal degradation. Indeed, HIF1*α* was abundant in nearly all oncocytic cells within the tumor mass compared to the normal tissue (Figure 2(a)-2(b)), confirming this to be a neoplasm with defective VHL. Nonetheless, HIF1*α* stabilization is usually not a feature of benign oncocytomas, which prompted us to investigate further how such a marker of malignancy may have occurred in this case. Staining for HIF1*α* target carbonic anhydrase IX (CAIX), used as an endogenous marker for tumor hypoxia in VHL-deficient renal cell carcinomas [8], surprisingly revealed negative cells throughout the mass (Figure 2(c)-2(d)). This result was also corroborated by the negative staining for GLUT1 in the renal oncocytoma (Figure 2(e)-2(f)), another well-known HIF1 target usually found to be positive in cancer hypoxic regions. The negative staining of both CAIX and GLUT1 was in apparent disagreement with the positive HIF1*α* staining, and it suggested that HIF1*α* was retained in its hydroxylated status and therefore lacking transcriptional activity.

### 3.3. Damage to the Mitochondrial Respiratory Chain May Underlie the Oncocytic Phenotype

Regardless of its abundance in the VHL tumor, a dysfunctional HIF1*α* was consistent with the oncocytic phenotype and its benign nature. In order to understand the cause for such lack of transcriptional activity despite its nuclear accumulation, we performed whole mitochondrial DNA sequencing with the aim to reveal pathogenic mutations that may impede a proper HIF1*α* functioning. The rationale for this approach was that mtDNA mutations hampering the activity of the respiratory chain trigger an imbalance of the NAD^+^/NADH ratio with consequent slowdown of the Krebs cycle rate. The latter may contribute to the accumulation of *α*-ketoglutarate, fostering the activity of PHDs [9]. In VHL-competent cells, PHDs would contribute to destabilize HIF1*α*, whereas in our VHL-deficient case we hypothesized that HIF1*α* should be still hyperhydroxylated, but not directed to the proteasome for degradation.

**Figure 3:**
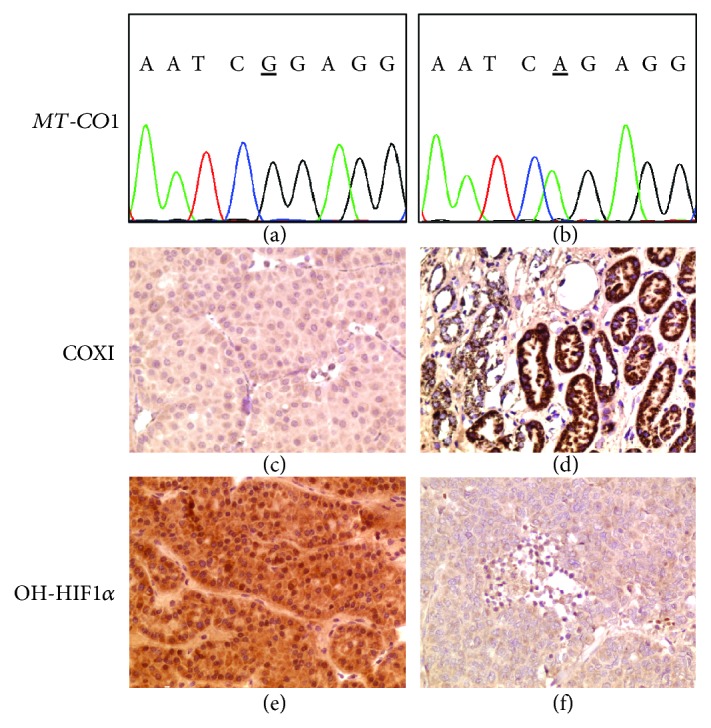
Genetic and IHC characterization of the renal oncocytoma from the patient carrying the germline *VHL* deletion. (a, b) Chromatograms showing a reference (a) and the tumor sequence with the somatic homoplasmic m.6129G>A stop-gain mutation found in *MT-CO1* (b). (c, d) IHC for COXI in the renal oncocytoma (c) with a QS of 0 (*P* = 0, *I* = 1) and in a normal kidney used as control (d) with a QS of 12 (*P* = 4, *I* = 3). (e, f) IHC for hydroxylated HIF1*α* (OH-HIF1*α*) in the renal oncocytoma (e) with a QS of 8 (*P* = 4, *I* = 2) and in the same hypoxic neuroendocrine carcinoma of the ovary stained for GLUT1 in Figure 2 used as control (f) with a QS of 0 (*P* = 0, *I* = 1). Magnification for all IHC images: 200x.

The screening of the tumor for mtDNA genetic lesions indeed revealed a stop-gain mutation in the *MT-CO1* gene (m.6129G>A) (Figure 3(a)-3(b)), encoding COXI, one of the three mtDNA subunits for respiratory CIV. The mutation was novel as it was not reported in the reference human mtDNA database HmtDB, http://www.hmtdb.uniba.it/hmdb/ [10], and it was strikingly homoplasmic. IHC staining for COXI was indeed negative in cancer cells (Figure 3(c)-3(d)), suggesting that CIV was not properly assembled.

Hence, we stained the renal oncocytoma for the hydroxylated form of HIF1*α* with the aim to understand whether the HIF1*α* positivity we observed (Figure 2(a)) was indeed due to the inactive form of the transcription factor, unable to be degraded in a VHL-deficient context. Indeed, a marked positive staining compared to control (Figure 3(e)-3(f)) indicated the accumulation of transcriptionally inactive hydroxylated HIF1*α*.

## 4. Discussion

Very few cases of renal oncocytoma occurring in the context of a VHL syndrome have been described [11, 12], underlining the extremely low prevalence of this type of benign neoplasm when *VHL* is mutated. This is likely due to the fact that the functional trigger for tumorigenesis in the presence of *VHL* mutations is HIF1*α* chronic stabilization, as loss of function mutations of the tumor suppressor lead to a lack of HIF1*α* ubiquitination and subsequent proteasome-mediated degradation. The latter, nonetheless, depends on a functional mitochondrial respiratory chain, since a block of activity allows both intracellular oxygen and NADH to accumulate, leading in turn to increased levels of *α*-ketoglutarate, feeding the PHD reaction [13]. In this clinical case, we detected a mtDNA mutation in CIV, which may have well triggered oncocytic transformation through, possibly, a compensatory effect to overcome the bioenergetic deficit. Such a severe homoplasmic mutation occurred in a bottleneck complex of the respiratory chain, whose negative staining strongly suggests that a severe energetic impairment may occur in these cells, hence explaining the indolent nature of the oncocytic neoplasm.

Therefore, on one hand the derangement of the respiratory chain would promote HIF1*α* hydroxylation and, on the other, the absence of VHL-mediated degradation would allow accumulation of inactive HIF1*α*. Hydroxylation on the HIF1*α* C-terminal activator domain impairs the interaction between HIF1*α* and the CBP/p300 coactivators, abolishing HIF1*α* transactivation activity [14]. Therefore, a severe dysfunction of the respiratory chain here may have favored the accumulation of high levels of hydroxylated HIF1*α* as shown by IHC. The low-functional HIF1*α* would not help to drive hypoxia response and to trigger the protumorigenic Warburg effect, as suggested by the negative CAIX and GLUT1 staining, a mechanism previously reported also as a result of respiratory Complex I disassembly and a dysfunctional cytochrome c [3, 15]. Along with a hampered respiratory chain, this would further contribute to maintaining the tumor in a low-proliferative and low-aggressive state by slowing down HIF1-dependent glycolysis; it is plausible to envision that this tumor may switch to the reductive carboxylation of glutamine to thrive, a metabolic route activated in the presence of severe mtDNA mutations [16]. Altogether, our data point to mtDNA mutations as potent modifiers of a cancer phenotype and fate. Further investigation of such mechanisms is warranted as targeted pharmacological derangement of the respiratory chain, i.e., through metformin [17, 18], may be useful as an adjuvant treatment for VHL tumors to dampen the effects of HIF1*α* chronic stabilization and hence tumorigenic potential.

## Data Availability

The mtDNA sequence of the oncocytic tumor described in this paper has been deposited in the Human Mitochondrial DataBase HmtDB [10] and may be retrieved with the following identifier: PA_EU_IT_0276.
